# Surface Properties of Poly(Hydroxyurethane)s Based on Five-Membered Bis-Cyclic Carbonate of Diglycidyl Ether of Bisphenol A

**DOI:** 10.3390/ma13225184

**Published:** 2020-11-17

**Authors:** Mariusz Tryznowski, Zuzanna Żołek-Tryznowska

**Affiliations:** Faculty of Production Engineering, Warsaw University of Technology, Narbutta 85, 02-524 Warsaw, Poland; zuzanna.tryznowska@pw.edu.pl

**Keywords:** poly(hydroxyurethane)s coatings, contact angle, surface free energy, adhesive

## Abstract

Poly(hydroxyurethane)s (PHU) are alternatives for conventional polyurethanes due to the use of bis-cyclic dicarbonates and diamines instead of harmful and toxic isocyanates. However, the surface properties of poly(hydroxyurethane)s are not well known. In this work, we focus on the analysis of the surface properties of poly(hydroxyurethane) coatings. Poly(hydroxyurethane)s were obtained by a catalyst-free method from commercially available carbonated diglycidyl ether of bisphenol A (Epidian 6 epoxy resins) and various diamines: ethylenediamine, trimethylenediamine, putrescine, hexamethylenediamine, 2,2,4(2,4,4)-trimethyl-1,6-hexanediamine, *m*-xylylenediamine, 1,8-diamino-3,6-dioxaoctane, 4,7,10-trioxa-1,13-tridecanediamine, and isophorone diamine, using a non-isocyanate route. The structures of the obtained polymers were confirmed by FT-IR, ^1^H NMR and ^13^C NMR spectroscopy, and thermogravimetric (TGA) and differential scanning calorimetry (DSC) analyses were performed. The rheological characteristic of the obtained polymers is presented. The static contact angles of water, diidomethane, and formamide, deposited on PHU coatings, were measured. From the measured contact angles, the surface free energy was calculated using two different approaches: Owens–Wendt and van Oss–Chaudhury–Good. Moreover, the wetting envelopes of PHU coatings were plotted, which enables the prediction of the wetting effect of various solvents. The results show that in the investigated coatings, a mainly dispersive interaction occurs.

## 1. Introduction

Knowledge of interfacial phenomena is necessary to understand the interaction and processes occurring at the surface and interface between two phases. Furthermore, this phenomenon is one of the most important in every industrial process including heterogeneous catalysis, manufacturing of composite materials, environmental protection, medical technology and finally technical processes, for example the fabrication of compound systems such as reinforced materials or coated materials [1]. The obtained adhesion between the layers is also influenced by proper surface preparation [2]. A suitable wettability promotes intimate adhesive-substrate contact, and it is an important factor that must be taken into consideration when adhesion can or cannot be achieved. Hence, the focus of intense scientific research is the wettability, surface free energy, and the properties of the surface layer. The contact angle (CA or *θ*) is allowed to assess the wettability of the solid by the liquid. It is well known that a wetting liquid forms a contact angle with the solid smaller than 90°; in contrast, a non-wetting liquid creates a contact angle larger than 90° [3]. Furthermore, with *θ* = 0°, drop spreads and complete wetting of the surface are observed. The surface free energy (SFE, γ) of solid surfaces is determined based on CA measurement. The SFE cannot be directly measured, however, there are many indirect methods that allow one to determine the surface free energy (SFE) of solids. However, the experimental data cannot be unambiguously interpreted with these approaches [4]. Generally, the methods depend on a measured values of static contact angle (CA) [5,6,7]. There are several methods of determination of SFE from CA data. However, the main methods are the Owens–Wendt [8] (OW, also known as the Owens–Wendt–Rabel–Kaelble method), and a relatively new method, the van Oss–Chaudhury–Good (vOCG) [9,10]. For polymeric materials, the OW method is mostly used. In this method, the static contact angle of two measuring liquids should be performed. Mainly water as polar and diiodomethane dispersive liquid is used. However, the vOCG method is the latest method and a subject of intensive research and scientific discussion. In the vOCG, three measuring liquids are used, mostly diiodomethane, water and formamide. The vOCG method, also called an acid-based approach, has the advantage of providing additional information in terms of acid and base components of SFE. To the best of our knowledge, the vOCG method has not been used for the determination of SFE of poly(hydroxyurethane) materials. The knowledge of SFE provides useful information on the several phenomena which occur at solid–liquid and solid-gas interfaces, like the adsorption mechanisms or the wetting behaviours.

It is well known that poly(hydroxyurethane)s (PHUs) are alternative to conventional polyurethanes (PU). The key reagents for conventional PU synthesis are polyols and harmful and toxic isocyanates, such as MDI (4,4′-diisocyanate) and TDI (toluene diisocyanate), which are classified as carcinogenic, mutagenic and reprotoxic [11,12]. The global productions of PUs reached 17 million tons in 2016 and they are ranked sixth among all polymers [13]. Furthermore, there is growing attention of researchers concentrated on replacing petroleum-derived chemicals with renewable sources or using less harmful PU, for example as waterborne polyurethane materials instead of solvent-based materials [14,15]. PHUs are not a novel group of polymers; many studies on their synthesis from bio-based renewable sources have already been presented in the literature [11,16,17,18,19,20,21,22,23,24,25,26,27,28,29,30,31,32]. Additionally, some hybrid PHUs have already been described [11,26,32,33,34,35]. Despite these polymers being known for nearly thirty years, their commercial application is limited to hybrid polyurethane manufactured without isocyanates, Green Polyurethane^TM^ (Nanotech Industries, Inc, Dala City, CA, USA) [18,36].

Anyhow, there are several problems with introducing PHU for large scale use. Firstly, the PHUs exhibit quite high hydrophobicity [31] and affinity to water, so their use is limited to applications which require a low humidity environment or even anhydrous conditions. The presence of pending hydroxyl groups in PHUs macromolecules is related to the much higher absorption of water in comparison to their PU analogues [37], and consequently causes the coating’s delamination [16], as well as changes in its properties. In contrast to conventional PUs, PHUs show exacerbated hydrophilia and water absorption compared to their PU analogues [38,39]. Hence, the water uptake of PHUs might affect the mechanical properties and at the same time is unfavourable for coating applications [40]. The improvement of water resistance is an important issue [40] because this property strongly limits the usage of PHUs coatings in technological application, particularly in those where they are directly exposed high humidity or even to water. The hydrophilic property of PHUs prevents the formation of a good quality joint between PHUs used as adhesives and rather hydrophobic plastics, such as PE [33]. Hence, several works deal with the problem of the hydrophilicity of PHUs [26,33,41,42,43], etc. For example, the incorporation of siloxane segments [42] or fluorinated moieties might increase the hydrophobicity of PHUs [26]. Secondly, there is a technological barrier of the synthesis of bis-cyclic carbonates as raw materials for the large scale production of PHU. For example, the direct synthesis of glycerol carbonate from CO_2_ and glycerol is, from the economic point of view, unprofitable [44], and additionally, it is non-feasible due to very low conversion because of the high stability of CO_2_ [45].

PHUs can be obtained by several routes [13,45,46]. However, the most important route is the polyaddition of five-membered cyclic dicarbonates and diamines [37,47,48,49]. The reaction of primary amines with cyclic carbonates is presently the most promising way to synthesize new urethanes without any harmful by-products. This reaction leads to macromolecules containing urethane bonding, as well as hydroxyl groups attached to primary (I), and secondary (II), carbon atoms. The pending hydroxyl groups affect the overall stability of the obtained compounds due to their ability to form intramolecular and intermolecular hydrogen bonds, and at the same time, lead the way for the subsequent modification of polymers [50]. The hydroxyl groups increase the water absorption and at the same time the chemical resistance and improve the adhesion [51]. Five-membered cyclic carbonates can be obtained by carbonation using dimethyl carbonate or carbon dioxide. Dimethyl carbonate is not harmful or mutagenic, but it is a flammable liquid. Additionally, the carbonation reaction by dimethyl carbonate is carried out at the boiling point of dimethyl carbonate. The reaction of epoxide rings with carbon dioxide enables the utilization of waste CO_2_. In our previous work, we have shown the possibility of using an epoxy resin based on diglycidyl ether of bisphenol A as a raw material for bis-cyclic dicarbonate synthesis [52].

To the best of our knowledge, the research focuses on the synthesis of new PHUs based on bio-sourced and ecological raw materials, rather than seeking their novel innovative applications. Hence, the novel applications of PHUs is not popular topic of scientific research. In general, PHUs exhibit lower molar masses and slower kinetics [13], so their application is limited by their properties. However, some of the possible uses of PHUs have been already presented, e.g., coatings [53,54], adhesives and glues [11,16,20,25,26,30,31,54,55] and foams [19]. It should be highlighted that the number of works describing the potential applications of PHUs is constantly growing.

**Scheme 1:**
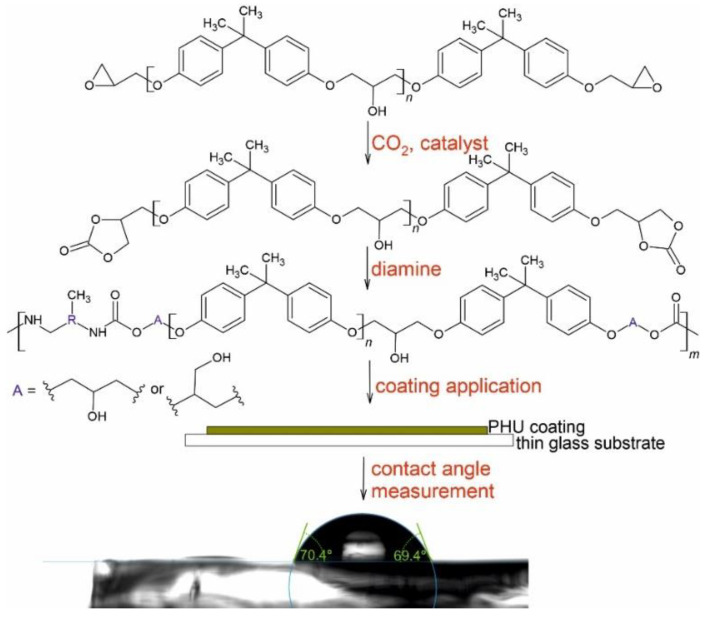
Synthesis of poly(hydroxyurethane)s (PHUs).

This work is a continuation of our research on improving the properties of PHU and their use as an adhesive or a coating [25,31,32,52,56,57]. The hanging off pendant hydroxyl groups in PHUs macromolecules strongly influence the hydrophilicity that limits the use of PHU coatings in technological applications. The goal of this study is focused on changes in the water sensitivity of PHUs. The novelty of this work is to use carbonated epoxy resin as a raw material changing the hydrophilicity of PHUs coatings. PHUs based on diglycydyl ether of Bisphenol A have been already presented in the literature [57]. In this work, instead of expensive diglycidyl ether of Bisphenol A, we have used commercially available and cheap epoxy resin, Epidian 6, as a raw material for PHUs synthesis. The epoxy resin, which is mainly a mixture of diglycidyl ether of bisphenol A, can be successfully used in the carbonation reaction with waste CO_2_, leading to five-membered bis-cyclic carbonates. The epoxy resin was used as received without any further purification or distillation. Last but not least, the epoxy resin is cheaper than diglycidyl ether of Bisphenol A. Then, to link the coating properties with the structure of the obtained PHUs, various diamines were used. The schematic workflow is shown in Scheme 1.

The structure of the obtained PHUs was confirmed by spectral analysis. We have shown the thermal and rheological properties of the obtained polymers. Moreover, we have determined the surface free energy of the obtained coatings by the Owens–Wendt, and additionally, by the van Oss–Good–Chaudhury methods, and finally, we have thoroughly analysed the wetting behaviour.

## 2. Materials and Methods

### 2.1. Materials

Epoxy resin, Epidian 6 (CAS 25068-38-6), was purchased from CIECH Sarzyna (Sarzyna, Poland) and use as-received. Ethylenediamine (purity ≥99%, CAS 107-15-3) was purchased from Avantor (former POCH, Gliwice, Poland). Trimethylenediamine (purity ≥99% CAS 109-76-2), putrescine (purity 99% CAS 110-60-1), hexamethylenediamine (purity 98% CAS 124-09-4), 2,2,4(2,4,4)-trimethyl-1,6-hexanediamine (purity 99% CAS 25620-58-0), m-xylylenediamine (purity 99% CAS 1477-55-0), 1,8-diamino-3,6-dioxaoctane (purity 98% CAS 2855-13-2), 4,7,10-trioxa-1,13-tridecanediamine (purity ≥99% CAS 2855-13-2) and isophorone diamine (purity ≥99% CAS 2855-13-2) were purchased from Sigma-Aldrich (Poznań, Poland). All reagents were used as received without further purification.

In order to perform contact angle measurements, diiodomethane (purity ≥99%, CAS 75-11-6) and formamide (purity ≥99%, CAS 75-12-7) purchased from Sigma-Aldrich (Poznań, Poland) were used. Distilled water was used as a polar liquid. 

Carbonated epoxy resin was obtained as described in our previous work [52].

### 2.2. Instrumentation

A Bruker ALPHA FT-IR (Billerica, MA, USA) spectrometer equipped with a Platinum single-reflection diamond ATR module was used to record FT-IR spectra in the wavelength range of 400–4000 cm^−1^, with a resolution of 4 cm^−1^.

^1^H NMR and ^13^C NMR spectra were measured with a Varian VXR 400 MHz spectrometer (Palo Alto, CA, USA). Tetramethylsilane as an internal standard and with deuterated solvents (DMSO-d_6_) was used. Obtained results were analyzed with MestReNova v.6.2.0 (Mestrelab Research S.L, Santiago de Compostela, Spain) software.

Basic thermal characteristics of the PHUs was measured with a differential scanning calorimetry (DSC) on TA Instruments Q2000 (New Castle, DE, USA) apparatus. A thermogravimetric analysis (TGA) was carried out with on TA Instruments SDT Q600 (New Castle, DE, USA) apparatus. The applied rate in both cases was 10 K min^−1^. Additionally, the weight loss was measured at a temperature of 150 °C as a function of time.

Polymer shear viscosity measurements were carried out with a Malvern Kinexus Pro rheometer (Malvern, England) equipped with parallel plate geometry (gap 0.4 mm using a spindle of diameter 10 mm). In the centre of the plate, a standard mass of the sample (0.3 g) was placed. The measurements were repeated at least twice with new samplings. The shear viscosity was in the specified temperature range (80–160 °C).

A K Paint Applicator RK Prints (Royston, UK) equipped with a 4-sided applicator and heated bed was used to prepare coatings on the cleaned glass substrates. The polymer and the glass substrate was heated up to 120 °C. A coating was prepared using a gap width of 120 μm and the wet film thickness provided was equal to half the gap size. The surface roughness of the coatings was controlled with gauge SRT-220 TestAn roughness meter (Gdańsk, Poland). The roughness value (Ra) was randomly measured 3 times on the surface of coatings prior wettability measurement. The measured surface roughness of the coatings was about 0.1 µm. Prior to the contact angle measurements, the prepared coatings were kept under an argon gas atmosphere.

Drop shape analysis system, DSA 30 (Krüss, Germany) was used to measure contact angle of smooth and horizontal sessile drops of the test liquids (water, diiodomethane and formamide) deposited on solid surface–PHU coatings. Needles with a diameter of 0.5 mm were used for all liquids. The contact angle was measured on the static drops 5 s after deposition. The contact angle was measured with the error ±3°. The drop shape analysis was performed using the tangent method 1. The reported contact angle values for liquids are the mean of six drops deposited on two separate coating samples. The measurements were performed under controlled environmental conditions of 23 °C and 50% relative humidity [58].

Statistical analysis was performed using a one-way analysis of variance, ANOVA. The statistical programme Statgraphics Centurion 18 (v.18.1.06 (StatPoint^®^, Inc., Warrenton, VA, USA) was used.

### 2.3. General Procedure for the Preparation of PHUs

PHUs were synthesized by the reaction of carbonated epoxy resin with diamine according to a well-known procedure reported elsewhere [28,57]. The formulation is listed in Table 1.

The 30 g of carbonated epoxy resin was dissolved in approx. 7 mL of DMSO in a 100 mL round bottom flask equipped with a mechanical stirrer and a nitrogen inlet. The amine was added after heating the mixture up to 100 °C. The reaction proceeded for 48 h. The FT-IR spectroscopy was used to monitor the reaction progress. The FT-IR, ^1^H NMR and ^13^C NMR results confirming the structure of the obtained PHUs are listed in Supplementary Materials.

## 3. Results and Discussion

### 3.1. PHUs Synthesis

The synthesis of PHUs was carried out using five-membered bis-cyclic dicarbonate obtained from epoxy resin and various diamines. In order to analyze the surface properties of the PHUs, various diamines were used. The synthesis of diglycerol dicarbonate from commercially available epoxy resin, Epidian 6 has been described in our previous work [52]. The formulations of PHU are listed in Table 1. The reaction was monitored by the FT-IR spectroscopy. During the reaction, the strongest peak at 1785 cm^−1^, assigned to the cyclic carbonate group, disappears. Next, new peaks typical of hydroxyurethane bonding appear (at 3312 cm^−1^, 1687 cm^−1^ and 1538 cm^−1^), which indicates the consumption of the bis-cyclic dicarbonate.

The chemical structures of the PHUs were further confirmed by ^1^H NMR and ^13^C NMR. Figures S1 and S2 in Supplementary Materials show the ^1^H NMR spectra of the monomer and PHU, respectively. The PHUs are not a new group of polymers, hence the detailed description of the PHU’s structure based on various bis-cyclic carbonates and diamines can be found elsewhere [32,59,60,61,62]. The urethane structure of the obtained PHU is confirmed by the signal in the range of 7.7 to 7.8 ppm from urethane proton, depending on the diamine used. A primary hydroxyl group is observed as a broad separate signal in the area of 5 ppm. The signal confirming the secondary hydroxyl group is hidden by the broad multiples in the range of 3.8–4.1 ppm. Furthermore, the presence of urethane groups (C=O) is confirmed by a signal at approx. 156 ppm in the ^13^C HNMR spectra.

Because of the lack of solubility of the polymers in typical solvents used in GPC technique, the molecular weight and molecular weight distributions were not determined.

### 3.2. Thermal Characterization

The results of TGA and DSC are summarized in Table 2.

The obtained polymers exhibited quite high values of the glass transition temperature *T*_g_, and the values are comparable with those shown previously for other PHUs, for example see in [63,64]. The highest value of *T*_g_ was observed for PHU-CC6 (115 °C) that is based on isophorone diamine; the lowest for PHU-C6O2 and PHU-C10O3 (36 °C and 34 °C, respectively). Furthermore, the glass transition temperature is higher than observed for the PHU-Ar based on *m*-xylylenediamine. The glass transition temperature for PHU-CC6 is also much higher than those observed for PHUs based on aliphatic diamine [56].

The obtained PHUs exhibited an initial degradation (at 5% weight loss) in the range of 239 °C to 283 °C and maximum decomposition rates in the range of 355 °C to 371 °C, values comparable to those observed previously by us [32,55,56]. The PHUs based on aliphatic diamines (NIPU1−NIPU3) exhibited the lowest temperatures of initial degradation (at 5% weight loss). Higher values of initial degradation temperature were observed for NIPU-Ar and PHU-C10O3. Furthermore, the lowest weight loss at maximum decomposition was observed for NIPU5. NIPU1 exhibited the highest thermal stability with maximum rate of degradation occurring at 411 °C, but simultaneously with the highest weight loss.

### 3.3. Rheological Properties

**Figure 1:**
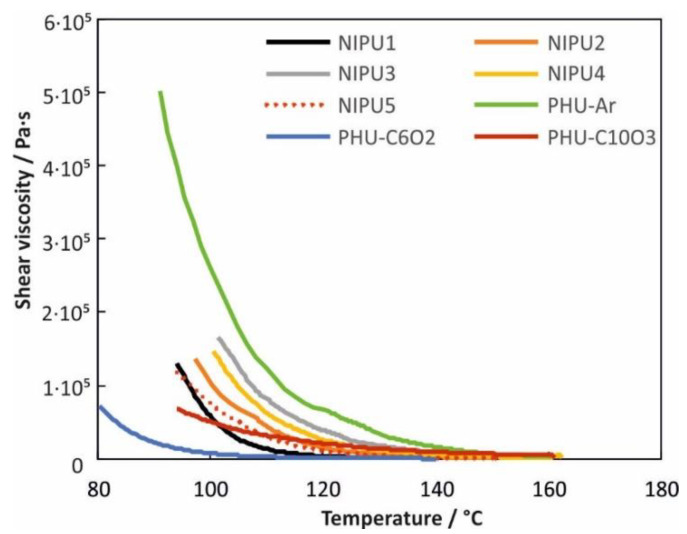
Shear viscosity of investigated poly(hydroxyurethane)s.

The temperature dependence of the shear viscosity of the obtained poly(hydroxyurethanes) is shown in Figure 1.

Due to the very high viscosity of PHU-CC6, we did not determine the viscosity of this PHU in the specified temperature range. The highest viscosity was observed by PHU-Ar and the lowest by PHU-C6O2 and PHU-C10O3, which is related with the presence of short rigid aromatic groups and flexible oxyethylene chains in the macromolecule, respectively. A similar tendency was observed by us, as noted in our previous work [32]. Hence, the shear viscosity of PHUs is mainly influenced by the properties of the diamine used, rather than the bis-cyclic carbonate. 

### 3.4. Wettability, Surface Free Energy

The determination of the static contact angles (CA) gives important knowledge regarding interactions between the coating and the liquid. Moreover, the absorption of water into the coating was observed, while the water contact angle measurements were performed. This effect was also observed for other NIPU layers [14,24,29] and it is directly related to the structure of the PHUs where pendant hydroxyl groups and urethane groups are built into the macromolecule. The pendant hydroxyl group improves the adhesion of PHUs with various substrates and influences the appearance of hydroxyl bonds [31].

In this work, to calculate SFE, two different methods were used: Owens–Wendt (also known as the Owens–Wendt–Rabel–Kaelble), and for the first time, van Oss–Good–Chaudhury.

The Owens–Wendt, OW, approach [8] was used to calculate the surface free energy of coatings, Equation (1):

(1) $$\left(1+\cos\theta_{i}\right)\gamma_{li}=2\left(\sqrt{\gamma_{li}^{d}\gamma_{s}^{d}}+\sqrt{\gamma_{li}^{p}\gamma_{s}^{p}}\right)$$

where *θ* is the contact angle (CA) of testing liquid (deg), *γ_li_* is the surface free energy (SFE) of the testing liquid $\gamma_{li}^{d}$,$\;\gamma_{li}^{p}$ are the dispersion and polar components of the tested liquid, and finally, $\gamma_{s}^{d}$,$\;\gamma_{s}^{p}\;$ are SFE of the coating.

The total surface free energy is the sum of polar and dispersive component according to Equation (2):

(2) $$\gamma_{s}=\gamma_{s}^{d}+\gamma_{s}^{p}$$

The acid base theory by van Oss, Good and Chaudhury, vOCG, was used for the determination of the dispersive, i.e., non-polar Lifshitz–van der Waals *γ*^LW^, acid (electron-acceptor) *γ*^+^, and base (electron donor) *γ^−^* components of the PHU surface energy [9,10], Equation (3):

(3) $$\left(1+\cos\theta_{i}\right)\gamma_{li}=2\left(\sqrt{\gamma_{i}^{\mathrm{LW}}\gamma_{s}^{\mathrm{LW}}}+\sqrt{\gamma_{i}^{\mathrm{LW}}\gamma_{s}^{\mathrm{LW}}}+\sqrt{\gamma_{i}^{\mathrm{LW}}\gamma_{s}^{\mathrm{LW}}}\right)$$

where *i* refers to the testing liquid and *s* refers to solid material.

The Lewis acid–base component was calculated from, Equation (4):

(4) $$\gamma_{s}^{\mathrm{LW}}=2\gamma_{s}^{+}\gamma_{s}^{-}$$

Moreover, the total surface free energy is defined by Equation (5):

(5) $$\gamma_{s}=\gamma_{s}^{\mathrm{AB}}+\gamma_{s}^{\mathrm{LW}}$$

The values of surface energy, its acid–base components and polar fraction of testing liquids used for OW and vOCG calculation are listed in Table 3.

The values of the contact angles of water, and the calculated SFE of various investigated PHU coatings, using the Owens–Wend and van Oss–Good–Chaudhury approaches, are summarized in Table 4.

Variance analysis of the results indicated a significant effect of PHU structure on contact angle measurements, as well as the calculated values of SFE and its components (*p*-value < 0.05).

The values of CA of water varied from 58 to 79° for PHU-C10O3 and NIPU5, respectively. Lower values of CA are related to the greater wettability and hydrophilicity of the surface. The values of CA decrease when the aliphatic chain in the diamine increases (from C2 to C4 in NIPU1 and NIPU 3, respectively), and the value of CA for water increases when the aliphatic chain in the diamine increases (from C4 to C5 in NIPU 3 and NIPU5). The same tendency was observed by us as shown in our previous work [56]. The CA of water is much lower for PHU-C6O2 and PHU-C10O3, which is related to the presence of oxygen atoms in the diamine chain.

**Figure 2:**
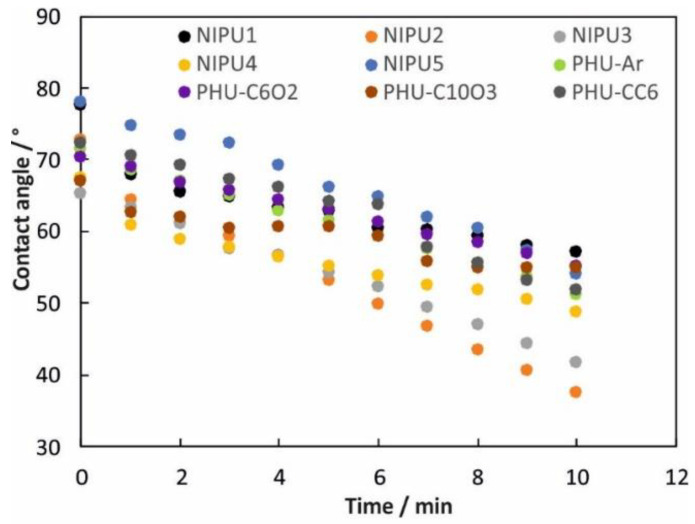
The changes of water contact angles over time.

The surface of PHU coatings is populated by polar groups such as urethane bonding, hydroxyl groups and additionally, depending on the PHU structure, oxygen atoms. These groups exhibit basic surface sites and can interact with the weakly polar H*^δ^*^+^ of the water molecule on the exterior. As a result, water absorption into the PHU coating surface is observed (see Figure 2).

As shown in Figure 2, the greatest water absorption was observed for NIPU2, whereas the CA decreases from 73° down to 38°. On the contrary, the lowest water absorption was observed for NIPU1 based on ethylodiamine, and PHU-CC6 and PHU-Ar based on isophorone diamine and *m*-xylylenediamine, respectively. These PHUs are more “hydrophobic” than the other obtained PHUs.

The values of total SFE determined with the Owens–Wendt approach are the highest for PHU-C10O3, equal to 57 mJ∙m^−2^; and the lowest value was calculated for PHU-CC6, equal to 47 mJ∙m^−2^. The values of total SFE are much lower than those calculated by us in our previous works [25,31,55]. The results show that in the investigated coatings, mainly dispersive interactions occur. The values of the polar component are very low, between 3 and 11 mJ∙m^−2^ for NIPU5 and PHU-C10O3, respectively. This indicates the poor polarity of the obtained PHUs, despite the presence of hydroxyl groups and other polar groups in the PHU molecules. However, the value of the polar part of PHU-C10O3 indicates a stronger polar character of this coating in comparison to the other investigated PHUs, which is related to the lowest value of water CA.

**Figure 3:**
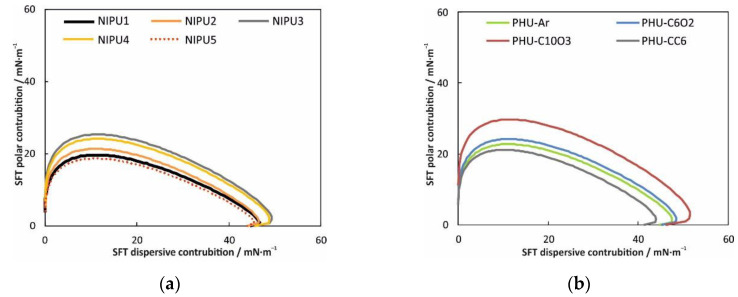
Wetting envelope contours for PHU coatings; the disperse (*γ*^d^) and the polar (*γ*^p^) components of the liquid surface tensions in mN/m are on the horizontal and the vertical axes, respectively. (**a**) NIPU1–NIPU5; (**b**) PHU-Ar, PHUC6O2, PHU-C10O3, PHU-CC6.

The knowledge of the polar and disperse parts of surface free energy allows us to calculate the curve known as the wetting envelope (Figure 3).

By plotting *γ*^p^ against *γ*^d^, a bow-shaped curve is obtained, which starts at the origin, reaches a maximum value, and then returns to the horizontal axis. If the contour of the wetting envelopes is known, the wettability of the solvent on the material can be predicted. The material is wetted with the contact angle of the wetting envelope, if the polar and dispersive parts of the liquid lie this curve [32]. Hence, the knowledge of wetting envelope curve allow to predict approximate value of contact angle without need for directly measurement.

For the first time, the SFE of PHU coatings was calculated using the van Oss, Chaudhury and Good method. This model has been known for nearly 30 years and is increasing in importance. However, to the best of our knowledge, there are no publications where the values of PHU SFE calculated with the vOCG approach have been presented. The values of total SFE determined with OW and vOCG approaches differ significantly. This tendency has already been observed in other works for various materials, for example PHU [31], wood [57], and plastics [7], where the values of SFE calculated with OW are slightly higher than those calculated by vOCG. The negligible difference between total SFE calculated with both approaches was observed for NIPU1; the greatest difference is for PHU-C6O2 and is equal to 6 mJ·m^−2^.

The Lifshitz–van der Waals component, *γ*^LW^, is responsible for non-polar interactions on the surface energy of a solid. The *γ*^LW^ surface energy component is a result of the London dispersion, the Debye dipole–dipole interaction, and the Keesom orientation [9]. The values of *γ*^LW^ are greater than the values of the acidic and basic part, *γ*^AB^, of SFE.

The values of *γ*^LW^ varied from 41 mJ·m^−2^ to 46 mJ·m^−2^ for PHU-CC6 and PHU-C10O3, respectively, and the values of *γ*^AB^ varied from 1 mJ·m^−2^ to 9 mJ·m^−2^ for NIPU5 and PHU-C10O3, respectively. Furthermore, the values of the basic components are higher than the values of the acidic components, because of the presence of electron pairs of oxygen and nitrogen atoms containing ether, carbonyl, and urethane parts of PHUs macromolecules. However, comparing the acidic components, it can be seen that *γ*^+^ gives the following tendency: NIPU1>NIPU2>NIPU3>NIPU4>NIPU5, so the *γ*^+^ increases with the increase of the alkyl chain in diamine, and NIPU1 exhibited the highest “acidic” surface character.

In conclusion, all investigated PHUs exhibited various values of *γ*^AB^ and *γ*^LW^ due to various amounts and types of functional groups (ether, carbonyl, and urethane) and free end-groups (hydroxyl) in the PHUs, regardless of their similar structure.

## 4. Conclusions

In this paper, the synthesis of non-isocyanate poly(hydroxyurethane)s based on carbonated epoxy resin and various amines and diamines is presented. The obtained PHUs were characterized by FT-IR, ^1^H NMR and ^13^C NMR spectroscopy and additionally by DSC and TGA.

The obtained PHUs were used as coatings. An extensive study on the surface properties of the PHU coatings was carried out. The surface free energies of the obtained PHU coatings were calculated using two different models: the well-known Owens–Wendt and additionally, for the first time, the van Oss, Chaudhury and Good. The values of contact angles and surface free energy and their disperse and polar components, as well as the Lifshitz–van der Waals and acid–base components, indicate solvent wettability. Moreover, the wetting envelopes of the PHU coatings were plotted, which allows the prediction of the wettability of various organic solvents. It should be highlighted that all the investigated PHU coatings shown water absorption—observed by us as a change in the water contact angle as a function of time—which is related to the great hydrophilic nature of the PHUs. However, the incorporation of hydrophobic chains in the diamine provides an easy way to modify the water affinity of the PHUs.

## Figures and Tables

**Table 1:** PHUs material formulation.

Name	Dicarbonate Mass (g)	Diamine	Diamine (mol)
NIPU1 ^1^	27.2	Ethylenediamine	0.0704
NIPU2 ^1^	28.3	Trimethylenediamine	0.0738
NIPU3 ^1^	26.0	Putrescine	0.0678
NIPU4 ^1^	27.2	Hexamethylenediamine	0.0710
NIPU5 ^1^	26.9	2,2,4(2,4,4)-Trimethyl-1,6-hexanediamine	0.0702
PHU-Ar	23.6	m-Xylylenediamine	0.0615
PHU-C6O2	28.1	1,8-Diamino-3,6-dioxaoctane	0.0732
PHU-C10O3	27.9	4,7,10-Trioxa-1,13-tridecanediamine	0.0729
PHU-CC6	26.8	Isophorone diamine	0.0693

^1^ the synthesis and some properties were presented in [57].

**Table 2:** Thermogravimetric (TGA) and differential scanning calorimetry (DSC) analyses of obtained PHUs.

Name	*T*_g_ (°C)	*T*_d,5%_ (°C)	*T*_d max_ (°C)	Weight Loss (%)
NIPU1 ^1^	70	243	411	78
NIPU2 ^1^	68	249	374	53
NIPU3 ^1^	58	243	367	55
NIPU4 ^1^	58	273	360	48
NIPU5 ^1^	61	283	324	25
PHU-Ar	71	283	355	48
PHU-C6O2	36	239	369	51
PHU-C10O3	34	284	370	55
PHU-CC6	115	269	371	49

^1^ the synthesis and some properties were presented in [57].

**Table 3:** Surface energy and its components (in mN∙m) of testing liquids used for surface free energy (SFE) calculation.

Name	*γ* ^D^	*γ* ^P^	*γ* ^T^	*γ* ^LW^	*γ* ^AB^	*γ* ^+^	*γ^−^*	*γ* ^T^
Water	35.3	3.7	38.9	38.6	3.8	0.5	3.2	42.4
Diiodomethane	33.1	4.1	37.3	36.7	4.8	0.0	5.7	41.5
Formamide	31.1	3.0	34.1	33.8	3.1	2.3	0.1	36.9

**Table 4:** Values of contact angle total surface free energy, its components determined by Owens–Wendt and van Oss–Good–Chaudhury method.

PHU	Contac Angle	Surface Free Energy (mJ∙m^−2^)							
		Owens-Wendt (dw) ^1^			van Oss–Chaudhury–Good (dfw) ^2^				
	Water	*γ* ^D^	*γ* ^P^	*γ* ^T^	*γ* ^LW^	*γ* ^AB^	*γ* ^+^	*γ^−^*	*γ* ^T^
NIPU1	77	45	4	48	45	4	2	2	48
NIPU2	73	44	5	49	44	5	2	4	49
NIPU3	66	46	8	53	46	5	1	10	51
NIPU4	68	46	7	52	46	2	1	11	48
NIPU5	79	44	3	47	44	1	0	6	45
PHU-Ar	71	45	6	50	45	4	1	8	48
PHU-C6O2	68	45	7	52	45	1	0	15	46
PHU-C10O3	58	46	11	57	46	10	1	31	55
PHU-CC6	73	41	6	47	41	2	0	13	43

^1^ based on CA of water and diiodomethane; ^2^ based on CA of diiodomethane, formamide and water.

## Supplementary Materials

The following are available online at https://www.mdpi.com/1996-1944/13/22/5184/s1, Figure S1: ^1^H NMR of monomer, Figure S2: ^1^H NMR of PHU compound, Table S1: PHUs structures.
