# Supplementary material for: Surface Properties of Poly(Hydroxyurethane)s Based on Five-Membered Bis-Cyclic Carbonate of Diglycidyl Ether of Bisphenol A
Source: Materials (Basel). 2020 Nov 17;13(22):5184. doi: 10.3390/ma13225184 (PMC7698458; doi:10.3390/ma13225184)
Supplement: Supplementary file 1 [file materials-13-05184-s001.pdf]

materials

Supplementary materials

Surface Properties of Poly(Hydroxyurethane)s based on Five-Membered Bis-Cyclic Carbonate of Diglycidyl Ether of Bisphenol A

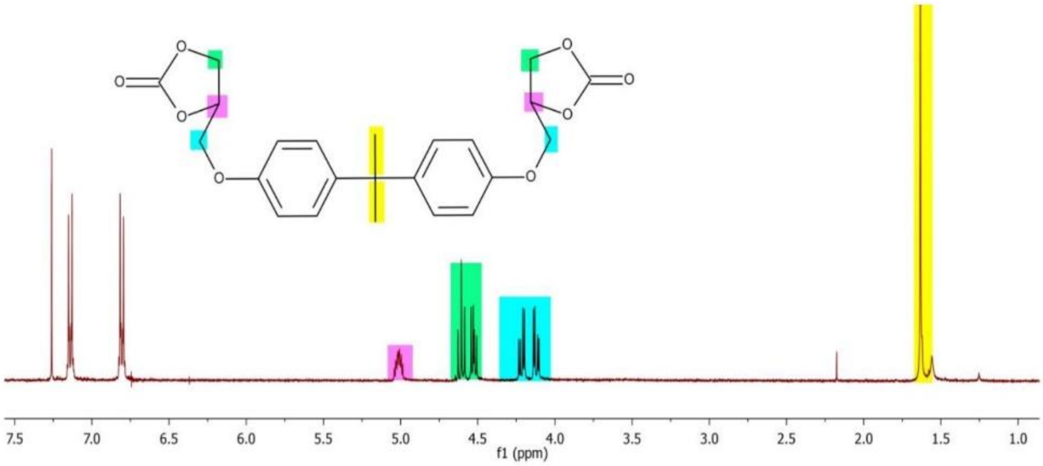

Figure S1. ^{1}H NMR of monomer.

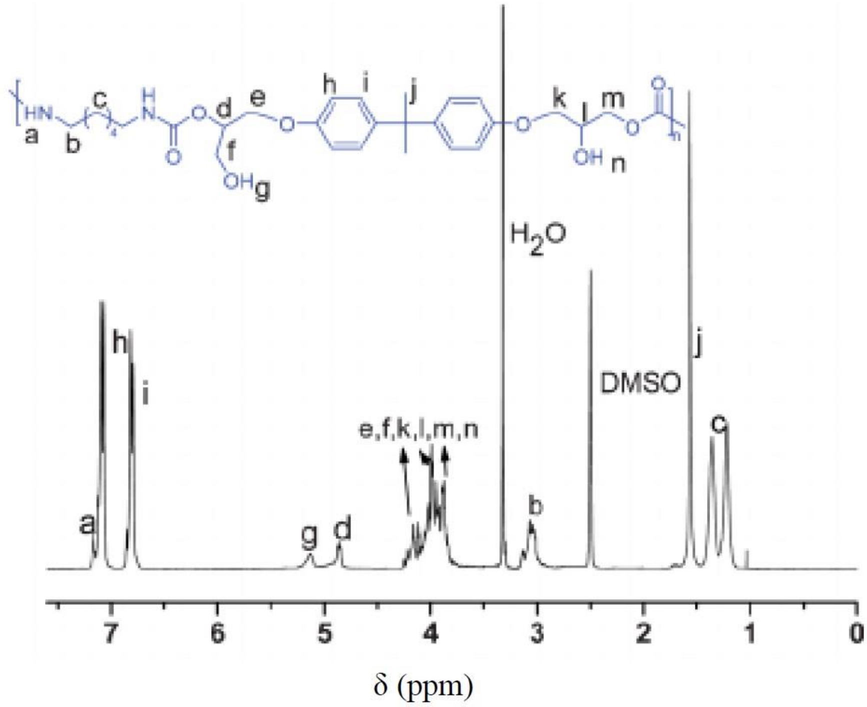

Figure S2. ^{1}H NMR of PHU compound.

Table S1. PHUs structures.

**PHU-Ar C**: ^{1}H NMR (400 MHz, DMSO-d_6); δ (ppm) = 7.82−7.71 (m, 2H), 7.11 (d, *J* = 8,3 Hz, 4H), 6.83 (d, *J* = 8.2, 4H), 7.05 (d, *J* = 8.4Hz, 2H), 6.76 (m, 1H), 6.70 (s, 1H), 5.27−5.20 (m, 1H), 5.00−4.85, (m, 1H), 4.10−3.82 (m, 10H), 3.60 (d, J=8.3 Hz, 4H), 1.57 (s, 6H). ^{13}C NMR (100 MHz, DMSO-d_6); δ (ppm) = 156.3 (C=O), 142.8, 128.3, 127.5, 125.8, 125.6, 125.0, 113.9, 72.8, 69.2, 67.3, 65.3, 59.8 (CH_2O and CHO), 55.0, 43.8, 40.4 (CH_2NH), 30.8; FT-IR (ATR) *υ* (cm^{-1}) 3330, 2963, 1695, 1607, 1506, 1457, 1361, 1226, 1181, 1130, 1036, 949, 828, 773, 555;
**PHU-C6O2 22**: ^{1}H NMR (400 MHz, DMSO-d_6); δ (ppm) = 7.24−7.14 (m, 2H), 7.08 (d, *J* = 8.3 Hz, 4H), 6.81 (d, *J* = 8.5 Hz, 4H), 5.27−5.22 (m, 1H), 4.99−4.81 (m, 1H), 4.05−3.82 (m, 10H), 3.55−3.44 (m, 4H), 3.10−3.05 (m, 4H), 2.66−2.60 (m, 4H), 1.54 (s, 6H). ^{13}C NMR (100 MHz, DMSO-d_6); δ (ppm) = 156.3, 142.7, 127.5, 113.9, 72.7, 69.5, 69.2, 67.3, 66.5, 66.2, 65.2, 59.8, 54.9, 41.2, 40.4, 30.8, 29.8; FT-IR (ATR) υ (cm^{-1}) 3330, 2929, 2870, 1697, 1607, 1507, 1459, 1361, 1231, 1182, 1102, 1036, 827, 773, 555;
**PHU-C10O3** 21: ^{1}H NMR (400 MHz, DMSO-d_6); δ (ppm)= 7.24−7.16 (m, 2H), 7.06 (d, *J* = 8.3 Hz, 4H), 6.82 (d, *J* = 8.5 Hz, 4H), 5.26−5,21 (m, 1H), 4.98−4.81 (m, 1H), 4.06−3.83 (m, 10H), 3.68−3.50 (m,8H) 3.58−3.44 (m, 4H), 3.07−3,00 (m, 4H) 2.56−2.48 (m, 4H), 1.54 (s, 6H). ^{13}C NMR (100 MHz, DMSO-d_6); δ (ppm) = 156.3, 142.7, 127.4, 113.9, 72.7, 69.5, 69.2, 67.2, 66.6, 66.1, 65.20, 59.7, 55.0, 41.2, 40.3, 30.8, 29.9; FT-IR (ATR) *υ* (cm^{-1}) 3336, 2921,34, 2870, 1695, 1607, 1507, 1457, 1361, 1234, 1182, 1039, 876, 828, 774, 555;
**PHU-CC6** 11: ^{1}H NMR (400 MHz, DMSO-d_6); δ (ppm) = 7.23−7.13 (m, 4H), 7.08 (d, *J* = 8.4 Hz, 4H), 6.80 (d, *J* = 8.2 Hz, 4H), 5.26−5.19 (m, 1H), 4.97−4.82 (m, 1H), 4.08−3.83 (m, 10H), 3.04−2.92 (m, 3H), 1.56 (s, 6H), 1.43 (d, *J* = 13.2 Hz, 2H), 1.30 (d, *J* = 13.1 Hz, 2H), 1.15 (d, *J* = 12,0 Hz, 3H) 1.10-1.00 (m, 2H), 0.99−0.89 (m, 1H), 0.84 (s, 3H); ^{13}C NMR (100 MHz, DMSO-d_6); δ (ppm) = 156.3, 142.7, 127.5, 113.9, 72.4, 69.2, 67.3, 65.1, 59.8, 54.4, 41.4, 41.2, 40.4, 35.0, 31.4, 30.8, 29.9, 27.5, 27.0, 23.2, 20.0; FT-IR (ATR) *υ* (cm^{-1}) 3334, 2954, 1698, 1607, 1507, 1460, 1385, 1362, 1229, 1181, 1032, 827, 772, 557.
